# Optimization of 16S RNA Sequencing and Evaluation of Metagenomic Analysis with Kraken 2 and KrakenUniq

**DOI:** 10.3390/diagnostics15172175

**Published:** 2025-08-27

**Authors:** Nasserdine Papa Mze, Cécile Fernand-Laurent, Sonnentrucker Maxence, Olfa Zanzouri, Solen Daugabel, Stéphanie Marque Juillet

**Affiliations:** 1 Service de Biologie, Unité de Microbiologie, Hôpital Mignot, Centre Hospitalier de Versailles, 177 rue de Versailles, 78150 Le Chesnay, France; cfernand@ght78sud.fr (C.F.-L.); ozanzouri@ght78sud.fr (O.Z.); sdaugabel@ght78sud.fr (S.D.); smarquejuillet@ght78sud.fr (S.M.J.); 2 Faculté de Santé, Université Paris-Est Créteil, 61 Avenue du Général de Gaulle, 94010 Créteil, France; maxencesonnentrucker@gmail.com

**Keywords:** kraken 2, krakenUniq, Smartgene, 16S RNA, Sanger, MiSeq

## Abstract

**Background/Objectives**: 16S ribosomal RNA sequencing has, for several years, been the main means of identifying bacterial and archaeal species. Low-throughput Sanger sequencing is often used for the detection and identification of microbial species, but this technique has several limitations. The use of high-throughput sequencers may be a good alternative to improve patient identification, especially for polyclonal infections and management. Kraken 2 and KrakenUniq are free, high-throughput tools providing a very rapid and accurate classification for metagenomic analyses. However, Kraken 2 can present false-positive results relative to KrakenUniq, which can be limiting in hospital settings requiring high levels of accuracy. The aim of this study was to establish an alternative next-generation sequencing technique to replace Sanger sequencing and to confirm that KrakenUniq is an excellent analysis tool that does not present false results relative to Kraken 2. **Methods**: DNA was extracted from reference bacterial samples for Laboratory Quality Controls (QCMDs) and the V2-V3 and V3-V4 regions of the 16S ribosomal gene were amplified. Amplified products were sequenced with the Illumina 16S Metagenomic Sequencing protocol with minor modifications to adapt and sequence an Illumina 16S library with a small 500-cycle nano-flow cell. The raw files (Fastq) were analyzed on a commercial Smartgene platform for comparison with Kraken 2 and KrakenUniq results. KrakenUniq was used with a standard bacterial database and with the 16S-specific Silva138, RDP11.5, and Greengenes 13.5 databases. **Results**: Seven of the eight (87.5%) QCMDs were correctly sequenced and identified by Sanger sequencing. The remaining QCMD, QCMD6, could not be identified through Sanger sequencing. All QCMDs were correctly sequenced and identified by MiSeq with the commercial Smartgene analysis platform. QCMD6 contained two bacteria, *Acinetobacter* and *Klebsiella*. KrakenUniq identification results were identical to those of Smartgene, whereas Kraken 2 yielded 25% false-positive results. **Conclusions**: If Sanger identification fails, MiSeq with a small nano-flow cell is a very good alternative for the identification of bacterial species. KrakenUniq is a free, fast, and easy-to-use tool for identifying and classifying bacterial infections.

## 1. Introduction

Bacterial infections are the second leading cause of death worldwide. In 2019, bacterial infections caused 13.7 million deaths around the world. Five of the thirty-three most common bacteria are responsible for half of all deaths: *Staphylococcus aureus*, *Escherichia coli*, *Streptococcus pneumoniae*, *Klebsiella pneumoniae*, and *Pseudomonas aeruginosa*. *Staphylococcus aureus* is the leading bacterial cause of death in 135 countries. In children under the age of five years, it causes the most deadly pneumococcal infection [1].

In France, incidence was significantly higher in 2022 than in 2021 for invasive infections caused by airborne and/or contact-transmitted bacteria: *Haemophilus influenzae*, *Neisseria meningitidis*, *Streptococcus pneumoniae*, and *Streptococcus pyogenes. Streptococcus pyogenes* was the most common species in children under 10 years of age [2].

Identification is traditionally based on the phenotypic characteristics of the bacterium: staining, morphology, ability to grow on certain culture media, and biochemical characteristics detected with various commercial techniques. However, it is difficult to identify some bacteria on the basis of phenotype, for various reasons [3,4,5,6]. Indeed, some bacteria express few phenotypic traits. In other species, stress may alter phenotypic characteristics [7]. Consequently, identification methods based exclusively on phenotypic characteristics can give erroneous results. Matrix-assisted laser desorption–ionization time of flight mass spectrometry (MALDI-TOF MS), a new technology applied to the problem of bacterial species identification, has been introduced in several microbiology laboratories [8]. However, although this technique offers an accurate identification approach, there may be limitations in the diagnosis of bacteria [9].

In such situations, amplification and sequencing of the 16S ribosomal RNA (rRNA) gene, followed by comparison of the resulting sequence with database sequences, have proven effective for bacterial identification [10,11,12,13]. The 16S rRNA gene encodes the 16S subunit of rRNA and has a structure that is highly conserved in all bacteria. It consists of a succession of conserved domains containing binding sites for the universal primers used to sequence this gene. The advantages of molecular biology techniques over traditional techniques include better sensitivity, significant time savings, and greater specificity for the identification of unusual bacteria in cultures.

Two sequencing techniques are available for bacterial identification: low-throughput analysis (Sanger sequencing) and high-throughput analysis (next-generation sequencing or NGS). Both methods analyze DNA at the single-base level, but they use very different approaches. Sanger sequencing generates fragments of the target sequence, each initiated from a primer. Each fragment ends with a fluorescent marker, with different colors corresponding to the nucleotides A, C, G, and T. These fragments are distinguished after separation via capillary electrophoresis. By contrast, NGS encompasses a wide range of techniques for sequencing DNA libraries. These libraries include vast collections of short DNA fragments, amplicons, or even mixed genome fragments from various species. In this high-throughput method, all of the sequences in a library are sequenced collectively. Unlike Sanger sequencing, NGS requires bioinformatics tools for the interpretation and analysis of the data obtained at the end of sequencing. Kraken was the first tool to introduce rapid taxonomic classification based on exact k-mer matches [14]. Kraken 2 constitutes a significant improvement in speed and memory while maintaining the classification algorithm of the original Kraken tool [15,16]. Kraken 2 uses a compact hash table and a probabilistic data structure, and it applies a spaced seed mask of s spaces to the minimizer and calculates a compact hash code [15]. It is generally superior for standard metagenomic analyses, but can have a low false-positive rate, making it less suitable for diagnosing infections [17,18]. KrakenUniq enhances Kraken by adding counts of unique k-mers for each classification, thereby providing a more accurate estimate of species abundance [19]. KrakenUniq uses the efficient cardinality estimation algorithm HyperLogLog for counting the number of unique k-mers identified for each taxon [20,21].

In this study, we sequenced the 16S gene with Sanger and NGS sequencing techniques and then used Kraken 2 and KrakenUniq to analyze the NGS-generated data. Several studies have shown that Kraken 2 is a very good tool for analyzing microorganisms, but very few studies have described its limitations [22,23,24]. The aim of this study was to set up an NGS technique as an alternative to Sanger sequencing and to confirm that KrakenUniq is an excellent bioinformatics analysis tool with a very low false-positive rate.

## 2. Materials and Methods

### 2.1. Validation Samples

Reference bacterial samples for quality control were obtained from the independent international organization Quality Control for Molecular Diagnostics (QCMD) in the form of supernatants from cultured reference bacterial strains. QCMD provides more than 2000 laboratories in more than 120 countries with a quality assessment service, with the aim of evaluating the ability of laboratories to detect, identify, and interpret the bacterial species supplied [25]. In addition to QCMD samples, we also included a positive and a negative control (DNA-free PCR).

### 2.2. Bacterial DNA Extraction

DNA was extracted with the EZ1 Virus Mini kit v2.0 (Qiagen S.A.S., Courtaboeuf, France) in accordance with the manufacturer’s instructions, but with an additional proteinase K pretreatment at 56 °C for 60 min.

### 2.3. Sanger PCR Amplification

Targeted PCR was performed on the V2/V3 region of the 16S rRNA with the following primers: 27F and 244R as previously described by Moumile et al. [26]. The PCR mixture contained 10 μL extracted DNA, 1 µL of each primer (12.5 μM), 1 µL dNTPs, 2 µL MgSO_4_, 5 µL 10 × buffer, and 0.2 µL Taq HIFI enzyme (Invitrogen, Carlsbad, CA, USA). We added 35 µL nuclease-free water to obtain a volume of 50 µL. PCR was performed under the following conditions: initial denaturation for 10 min at 94 °C, followed by 35 cycles of 30 s at 94 °C, 30 s at 58 °C, 30 s at 68 °C, and a final extension for 7 min at 68 °C. The amplified products were subjected to quality control via agarose gel electrophoresis. The amplicon obtained was about 357 base pairs (bp) long.

### 2.4. MiSeqPCR Amplification

Targeted PCR was performed on the V3/V4 region of the 16S RNA with primers containing Illumina adapter sequences, 341F and 785R, as previously described [27]. The reaction mixture contained 12.5 µL KAPA HiFi DNA polymerase (Reference KK2601, Roche, Cape, South Africa), 0.5 µL of each primer, 1.5 µL nuclease-free water, and 10 µL extracted DNA. The following PCR conditions were used: initial denaturation for 3 min at 95 °C, followed by 45 cycles of 30 s at 95 °C, 30 s at 55 °C, 30 s at 72 °C, and a final extension for 5 min at 72 °C. Amplified products of 550 bp in size were detected on migration in a TapeStation system.

### 2.5. Sanger Library Sequencing

Amplified products were first purified with the Monarch^®^ Spin PCR & DNA Cleanup Kit (Biolabs, Newburyport, MA, USA) according to the manufacturer’s instructions. Sequencing was then performed with the Big Dye Terminator mix. The reaction mixture consisted of 1 µL of the sense and antisense primers used in the first amplification, 13 µL nuclease-free water, 2 µL purified DNA, and 4 µL Big Dye Terminator mix. PCR was performed with 30 cycles of 10 s at 96 °C, 10 s at 50 °C, and 4 min at 60 °C. At the end of the reaction, ddNTPs were removed with rehydrated Sephadex G50 (Cytiva, Uppsala, Sweden), as recommended by the manufacturer. The purified products were then sequenced on a Thermo Fisher ABI 3500 sequencer (Thermo Fisher, Waltham, MA, USA).

### 2.6. MiSeqLibrary Sequencing

The library was prepared with a slightly modified and adapted version of the Illumina 16S Metagenomic Sequencing protocol (Part # 15044223 Rev. B). PCR products were purified with 0.8× AMPure XP beads and then diluted to 3.5 ng/µL. An index PCR was then performed on a mixture containing 25 µL KAPA HiFi mix (Beckman, CA, USA), 5 µL of each Nextra XT index (Illumina, San Diego, CA, USA), and 15 µL purified DNA. PCR conditions were as follows: initial denaturation for 3 min at 95 °C, followed by 12 cycles of 30 s at 95 °C, 30 s at 55 °C, 30 s at 72 °C, and a final extension for 5 min at 72 °C. After this index PCR, a second purification was performed with 1.2× magnetic beads. Each amplicon was diluted to a final concentration of 2 nM. All amplicons were pooled in a single tube, and 5 µL of this pool was denatured with 0.2 NaOH and diluted to 8 pM in HT1. The library was sequenced with a MiSeq Nano reagent kit v2, 500 cycles (Illumina Inc., San Diego, CA, USA).

### 2.7. Analysis Results

For Sanger sequencing, we used the sequences for a BLAST+2.16.0 analysis of the NCBI database to identify the various bacteria. By contrast, for MiSeq, we used three methods for bioinformatics analysis: the Advanced Sequencing Platform (ASP) v3.15.0 available from SmartGene (SmartGene, Unterägeri, Switzerland, www.smartgene.com (accessed on 1 May 2025)) [28], Kraken 2, and KrakenUniq.

The SmartGene application was used as a validation method. The SmartGene Bacteria 16S Microbiome App is a commercial CE-IVD-labeled automated cloud application service that can handle base-called sequencing files generated by different sequencing technologies. *.fastq files obtained with the MiSeq platform (Illumina) were uploaded into ASP. The application functions as follows. It first performs the following steps in an automated manner:

(1) Paired-end detection of read files, if uploaded in the same batch;
(2) Technology-specific quality filtering to trim or remove low-quality reads;
(3) Establishment of a work list for the batch.

The user then selects the analysis pipeline to be used, in this case, the Microbiome 16S targeted workflow. This automated analysis pipeline performs the following actions:

(1) Quality filtering: R1 and R2 files are merged for Illumina data, and a sliding window (25 nt) is then applied to reads, enhancing the trimming of poor-quality sections with a low Phred score (<23 for this study) and the filtering of short reads (<20 nt in this study).
(2) Read mapping: Reads passing the quality filters are mapped against the SmartGene 16S Centroid database without prior binning. The SmartGene “16S Centroid” database consists of non-redundant representative bacterial 16S rRNA sequences covering 17,314 species from 3439 genera as of November 2024. It is maintained and updated by AI and algorithms (patent #EP02215578).
(3) Production of a quality report and display of the results: Results are grouped according to match quality (e.g., % coverage, number of mismatches, etc.), match specificity (matching a single species or not), and match consistency (close matches belonging to the same genus). The system produces a confidence score for the matching taxon, and it is not possible to provide a call at the species level; a call is made at the next taxon up, indicating all possible matching species and genera. Results are displayed in table format, along with the number of reads and relative abundance, with the possibility of consolidation at species, genus, and family levels, and the generation of a dynamic Krona diagram. Abundances are evaluated by counting the reads mapped to a particular species/genus/family.

**Figure 1:**
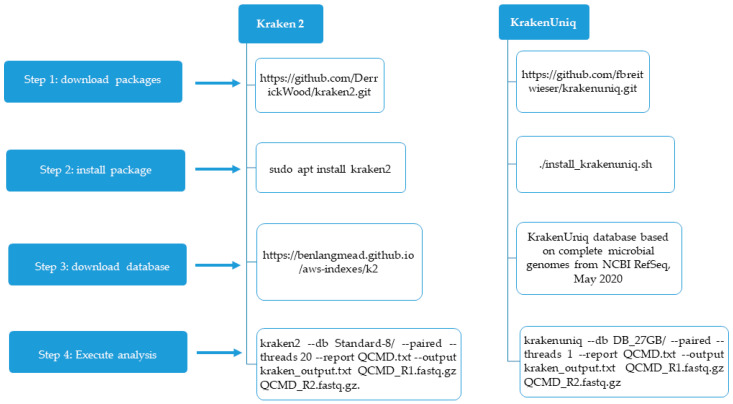
The different steps for configuring Kraken 2 and KrakenUniq.

Kraken 2 and KrakenUniq were installed on a 64-bit Linux computer with 8 GB RAM. The Kraken 2 package was downloaded as a Zip file from https://github.com/DerrickWood/kraken2.git (accessed on 1 May 2025) (Figure 1).

This file was unzipped and the following command line was used to install Kraken 2: sudo apt install kraken 2. For the running of Kraken 2, we downloaded an 8 GB (Standard-8) database from https://benlangmead.github.io/aws-indexes/k2. (accessed on 2 May 2025). We used the following command line to analyze each sample: kraken2 --db Standard-8/ --paired --threads 20 --report QCMD.txt --output kraken_output.txt QCMD_R1.fastq.gz QCMD_R2.fastq.gz. In this command line, Standard-8 is the database, and QCMD.txt is the file containing the classification results from the fastq.gz files.

We also used specific 16S databases—the SILVA database, Greengenes 13.5, and the RDP 11.5 database—for the Kraken2 analysis. These databases are all available from https://benlangmead.github.io/aws-indexes/k2. (accessed on 2 May 2025).

KrakenUniq was downloaded from https://github.com/fbreitwieser/krakenuniq.git (accessed on 2 May 2025) and installed with the following command line: ./install_krakenuniq.sh.

We downloaded an indexed 27 G database by clicking on the following link: KrakenUniq database based on complete microbial genomes from NCBI RefSeq, May 2020.

We used the following command line for the analysis of each sample: krakenuniq --db DB_27 GB/ --paired --threads 1 --report QCMD.txt --output kraken_output.txt QCMD_R1.fastq.gz QCMD_R2.fastq.gz.

## 3. Results

In total, eight QCMDs were subjected to Sanger sequencing and sequencing on a MiSeq system. Seven of these QCMDs (87.5%) were correctly sequenced through Sanger sequencing and were analyzed with NCBI Nucleotide Blast (Table 1). Total score and percent identity (Per. Indent) ranged from 963% to 11,245% and from 99.55% to 100%, respectively. Each QCMD was identified with a single species different from the others. The results of QCMD6 were not interpretable through Sanger sequencing, as this QCMD is a mixture of two microbes.

**Figure 2:**
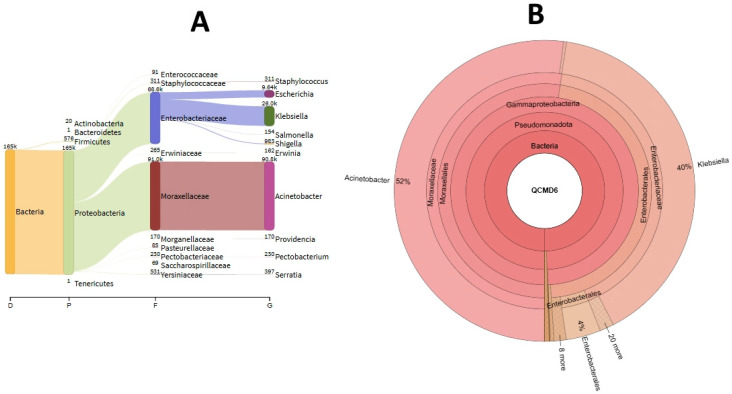
KrakenUniq (**A**) and Smartgene (**B**) analyses.

All eight QCMDs (100%) were correctly sequenced by MiSeq. The number of reads mapped with the Smartgene analysis application ranged from 116,822 (95.77%) to 188,722 (95%) (Table 2). Two genera were identified for QCMD6—*Acinetobacter and Klebsiella*—accounting for 52.02% and 40.09% of reads, respectively (Figure 2A). The mean analysis time for the eight QCMDs was 4080 s.

The number of reads mapped with KrakenUniq ranged from 117,091 (99.28%) to 189,218 (99.5%). *Acinetobacter* and *Klebsiella* were also identified in QCMD6 and these two genera accounted for 69.87% and 20% of reads, respectively, according to KrakenUniq (Figure 2B). The mean analysis time for the eight QCMDs was 27 s using KrakenUniq. No false positives were observed with Smartgene and KrakenUniq.

**Figure 3:**
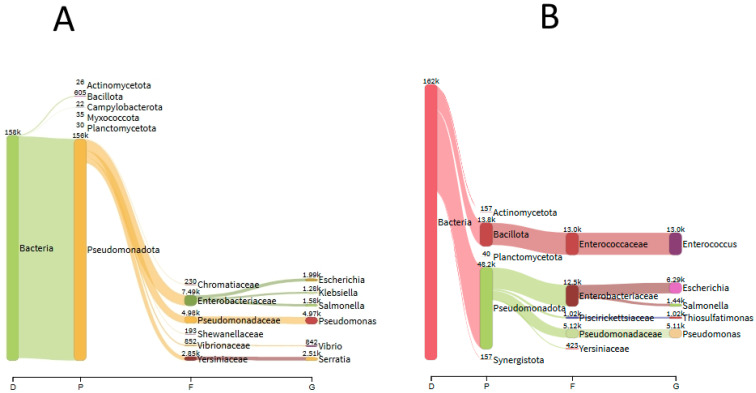
Kraken 2 analysis for QCMD1 (**A**) and QCMD5 (**B**).

By contrast, Kraken 2 analysis with the standard database presented 25% (2 of 8 QCMDs) false positives, including QCMD1, identified as *Pseudomonas* with 158,379 reads (99.26%), and QCMD5, identified as *Enterococcus* with a total of 165,385 reads (99.83%) (Figure 3). Conversely, the mixture of bacteria in QCMD6 was correctly identified with 21.21% *Acinetobacter* and 25.14% *Klebsiella* reads. The mean analysis time was 27 s.

An analysis of the Kraken 2 results obtained with the 16S-specific databases also revealed several false-positive results (Table 3). In the Silva138 database, QCMD2 was identified as *Streptococcus* with a total of 13,598 reads (34.97%), and QCMD7 was identified as a mixture of two bacteria: *Enterobacter* with 7798 (25.6%) reads and *Klebsiella* with 7060 (23.5%) reads. Only one of the microbes present in QCMD6, *Acinetobacter*, was correctly identified, with 85,118 reads (83.59%), despite the mixed nature of the infection, with both *Acinetobacter* and *Klebsiella* present.

The Greengenes database gave incorrect results for QCMD1 and QCMD5, both of which were identified as *Serratia*, with 17,029 (72.03%) and 1694 (11.23%) reads, respectively. The mixed infection in QCMD6 was not correctly detected, as only *Acinetobacter* was identified, with a total of 55,180 reads (86.58%).

For the RDP11.5 database, QCMD1 was also identified as *Serratia*, with 90,122 (93.03%) reads. QCMD7 was identified as a mixture of two bacteria, *Klebsiella* with 5054 (29.05%) reads and *Enterobacter* with 3287 (18.9%) reads. The QCMD6 was identified as a single bacterium, *Acinetobacter*, with 23,369 (69.72%) reads.

The total time for library preparation was 3 h 8 min for MiSeq and 4 h 45 min for Sanger. The sequencing time was 8 h for MiSeq and 1 h 30 min for Sanger.

## 4. Discussion

For over 35 years, clinical microbiology laboratories have used targeted sequencing for the definitive identification of bacterial pathogens of humans [29,30,31,32]. The 16S rRNA gene is a slowly evolving, highly conserved gene found in all microorganisms, features that have led to it becoming the most widely used target for bacterial and archaeal pathogen identification studies [33,34,35,36,37]. In this study, we used Sanger 16S sequencing technology to detect and identify various pathogens. This technique is often used when attempts to identify or detect bacteria in clinical samples are unsuccessful. However, this technique is unsuitable for the simultaneous detection of more than one species in a single sample and is, therefore, of limited value for use in cases of polymicrobial infection [38]. This limitation is illustrated by our results for QCMD6, corresponding to a polymicrobial infection with two species, *Acinetobacter baumannii* and *Klebsiella pneumoniae*. Despite this limitation, the rest of the samples (85.7%) were successfully identified through Sanger sequencing. The Sanger technique remains a rapid, cost-effective, high-performance method for detecting monomicrobial infections.

**Figure 4:**
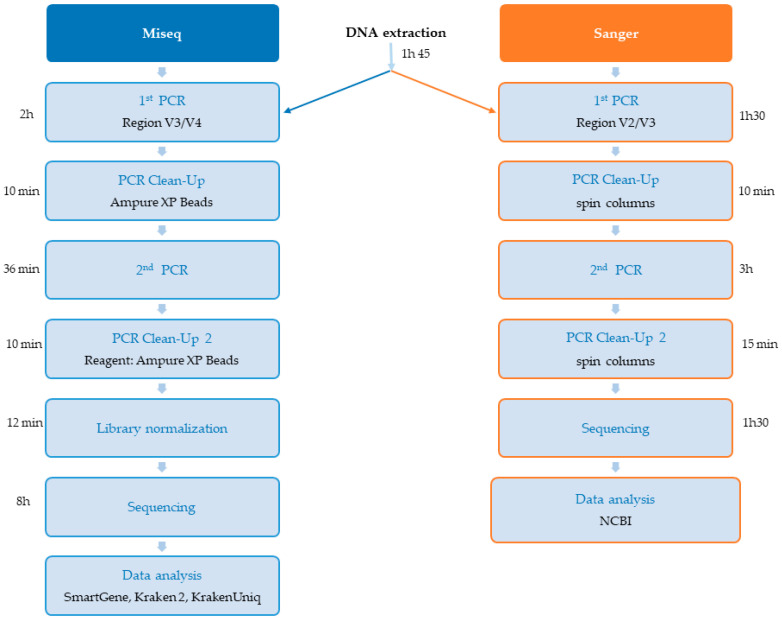
Sequencing times with MiSeq and Sanger.

As a means of overcoming the problem of bacterial identification in polymicrobial infections, we optimized and adapted the Illumina 16S protocol for the rapid sequencing of samples and bacterial identification within 24 h. The recent arrival of an Illumina MiSeq sequencer in our CHV laboratory [39] has opened up new possibilities for 16S sequencing, including the use of a nano-flow cell for the sequencing of a small number of samples with about one million reads. This new technique is highly suitable for use in clinical practice, in which results for individual patients must be obtained rapidly to optimize treatment. The results obtained with the Smartgene analysis platform were entirely concordant with those obtained through Sanger sequencing for the identification of bacteria in monomicrobial infections. Polymicrobial infection was correctly identified and differentiated. We chose to use this commercial Smartgene analysis platform because it was specifically developed to describe the composition of microbiota [28]. It is, thus, suitable for use to identify mixed (polymicrobial) infections and minority species. With this commercial platform, we were able to validate the quality of reads (Q30 ≥ 89% for all samples) obtained through sequencing and to validate the other analysis tools (Kraken 2 and KrakenUniq) assessed in this study. An analysis performed after sequencing yielded a Q30 value ≥ 89% and a Clusters Passing Filter rate ≥ 92%, demonstrating successful sequencing with a very good read quality. The technique used for library preparation is faster (libraries for eight samples prepared in about 3 h and 10 min, Figure 4) than other technologies requiring an additional fragmentation step. Furthermore, this protocol does not require the purchase of a specific library kit (just the KAPA HiFi HotStart ReadyMix with Illumina indices). This technique therefore remains a very good alternative approach to the identification of bacteria based on 16S RNA sequences for NGS.

Kraken 2 and KrakenUniq are free analysis tools from Kraken, the first tool for very fast taxonomic classification based on exact k-mer matches. The number of reads mapped for all samples was similar for Smartgene and Kraken 2 (standard database), but false positive results were obtained for QCMD1 and QCMD5. We also tested Kraken 2 with all of the available 16S-specific databases (Silva, Greengenes, and RDP), as in several other studies [40,41,42,43,44,45], with the aim of identifying a database giving no false positive detections. Unfortunately, all Kraken 2 databases gave at least 14.2% false positives for monomicrobial infections, and polymicrobial infection was not correctly identified with any of the 16S-specific databases. Thus, regardless of the database used, Kraken 2 is less suitable for the diagnosis of infections in hospital settings, in which a high degree of precision is required.

The KrakenUniq analysis yielded results comparable to those obtained with Smartgene. No false positives were observed, and the polymicrobial infection (QCMD6) was correctly identified. This study thus provides the first demonstration of practical cases of false positives with Kraken 2 analysis being corrected by KrakenUniq analysis. In addition to its simplicity of execution and speed of analysis (mean of 27 s per sample), KrakenUniq is clearly highly suitable for use in mono- and polymicrobial infections. We show herein how it is possible to install and run bioinformatics analyses without any specific prerequisite knowledge, on a low-capacity 8G RAM computer, as reported by C. Pockrandt et al. 2022 [45], to obtain reliable and robust analysis results. Furthermore, these analyses with KrakenUniq can be performed locally, without the need for Internet or a specific network.

Encouragingly, MiSeq sequencing successfully resolved all of the cases for which Sanger sequencing had yielded uninterpretable results in the last five years. In total, 32 clinical samples were sequenced on the MiSeq system and analyzed with KrakenUniq (unpublished data). Eleven of these samples (35%) were identified as polymicrobial infections, with the remainder being identified as monomicrobial. These results indicate that there are very few polymicrobial infections in our study (7% per year on average). The inability of Sanger sequencing to identify certain monomicrobial infections may be due to a low DNA concentration [46] following prior antibiotic treatment. As a means of ensuring better patient care, we will, from now on, systematically subject samples for which Sanger sequencing is unsuccessful to sequencing on the MiSeq sequencer. We strongly recommend the use of the 16S Illumina library preparation technique and KrakenUniq in all laboratories involved in microbial diagnosis.

## 5. Conclusions

This study confirms that KrakenUniq is a good bioinformatics analysis tool, similar to Kraken 2 in that it yields no false positives. Furthermore, it is free, easy to use, and very fast. Sanger sequencing remains cheaper and faster than NGS, but the use of a nano-flow cell on a MiSeq system remains a good alternative and the best solution for identifying polymicrobial infections while maintaining a reasonable turnaround time to ensure optimal patient care.

## Figures and Tables

**Table 1:** Sanger sequencing results.

Methods		Sanger/NCBI			
Sample	Max Score	Total Score	% Query Cover	% Per.Ident	Species
QCMD1	1218	1218	100	99.55	*Serratia mancesens*
QCMD2	1358	8128	100	99.86	*Enterococcus faecium*
QCMD3	1264	1264	100	99.85	*Staphylococcus aureus*
QCMD4	1465	1465	100	100	*Staphylococcus epidermidis*
QCMD5	963	963	99	99.62	*Escherichia coli*
QCMD6	ND	ND	ND	ND	ND
QCMD7	1423	11,245	100	99.87	*Klebsiella pneumoniae*
QCMD8	1589	1589	100	99.77	*Acinetobacter baumanii*

ND, not determined.

**Table 2:** Results of Smartgene, KrakenUniq, and Kraken 2 analyses with the standard database.

Methods	Sample	Mapped Reads	% Mapped Reads	% Reads Genus	Genus
Smartgene	QCMD1	156,342	93.3	93.66	*Serratia*
	QCMD2	141,434	95.65	95.3	*Enterococcus*
	QCMD3	188,746	95	98.6	*Staphylococcus*
	QCMD4	116,822	95.77	98.46	*Staphylococcus*
	QCMD5	160,715	93.82	95.95	*Escherichia*
	QCMD6	165,592	93.5	52.02	*Acinetobacter*
				40.09	*Klebsiella*
	QCMD7	157,750	93.7	87.57	*Klebsiella*
	QCMD8	123,272	92	97.65	*Aceinetobacter*
KrakenUniq	QCMD1	158,363	99.25	97.64	*Serratia*
	QCMD2	141,513	99.52	97.48	*Enterococcus*
	QCMD3	189,218	99.5	99.17	*Staphylococcus*
	QCMD4	117,091	99.28	99.11	*Staphylococcus*
	QCMD5	162,476	99.56	89.4	*Escherichia*
	QCMD6	165,392	99.83	69.87	*Acinetobacter*
				20	*Klebsiella*
	QCMD7	158,227	99.67	65.75	*Klebsiella*
	QCMD8	123,647	99.77	98.92	*Acinetobacter*
Kraken 2	QCMD1	158,376	99.26	28.27	*Pseudomonas*
	QCMD2	141,520	99.53	67.79	*Enterococcus*
	QCMD3	189,263	99.52	55.45	*Staphylococcus*
	QCMD4	117,149	99.33	56.66	*Staphylococcus*
	QCMD5	162,405	99.51	39.65	*Enterococcus*
	QCMD6	165,385	99.83	25.14	*Klebsiella*
				21.21	*Acinetobacter*
	QCMD7	158,256	99.69	32.56	*Klebsiella*
	QCMD8	123,671	99.79	60.25	*Acinetobacter*

**Table 3:** Kraken 2 results with 16S-specific databases.

Database	Samples	Total Read	Mapped Reads	% Mapped Reads	Coverage Domain	Coverage Phylum	Reads Genus	% Reads Genus	Genus
SILVA138	QCMD1	159,562	156,959	98.37	99.99	99.99	88,649	86.78	*Serratia*
	QCMD2	142,190	141,501	99.52	100	99.22	13,598	34.97	*Streptococcus*
							12,248	31.5	*Enterococcus*
	QCMD3	190,175	189,271	99.52	100	99.99	76,282	81.38	*Staphylococcus*
	QCMD4	117,944	117,128	99.31	99.99	99.99	46,259	81.26	*Staphylococcus*
	QCMD5	163,198	161,758	99.12	99.99	100	34,210	50.31	*Escherichia–Shigella*
	QCMD6	165,669	165,240	99.74	99.99	99.99	85,118	83.59	*Acinetobacter*
	QCMD7	158,745	157,643	99.31	100	99.99	7798	25.6	*Enterobacter*
							7060	23.18	*Klebsiella*
	QCMD8	123,933	123,558	99.7	99.99	100	119,341	98.34	*Acinetobacter*
Greengenes 13.5	QCMD1	159,562	156,917	98.34	99.99	99.99	17,029	72.03	*Serratia*
	QCMD2	142,190	141,477	99.5	99.99	100	13,146	51.8	*Enterococcus*
	QCMD3	190,175	189,236	99.51	99.99	100	75,474	94.42	*Staphylococcus*
	QCMD4	117,944	117,103	99.29	100	99.99	44,765	94.22	*Staphylococcus*
	QCMD5	163,198	161,725	99.1	100	100	1694	11.23	*Serratia*
	QCMD6	165,669	165,223	99.73	100	99.99	55,180	86.58	*Acinetobacter*
							4100	6.433	*Klebsiella*
	QCMD7	158,745	157,627	99.3	99.99	100	8492	49.97	*Klebsiella*
	QCMD8	123,933	123,552	99.69	100	100	78,297	98.75	*Acinetobacter*
RDP 11.5	QCMD1	159,562	156,925	98.35	100	99.99	90,122	93.07	*Serratia*
	QCMD2	142,190	141,486	99.5	100	100	10,255	38.52	*Enterococcus*
	QCMD3	190,175	189,245	99.51	99.99	100	48,119	94.91	*Staphylococcus*
	QCMD4	117,944	117,106	99.29	100	100	28,241	94.71	*Staphylococcus*
	QCMD5	163,198	161,735	99.1	100	100	26,615	85.38	*Escherichia–Shigella*
	QCMD6	165,669	165,226	99.73	99.99	100	23,369	69.72	*Acinetobacter*
	QCMD7	158,745	157,636	99.3	99.99	100	5054	29.05	*Klebsiella*
					99.99	100	3287	18.9	*Enterobacter*
	QCMD8	123,233	123,558	99.7	99.99	100	33,037	92.47	*Acinetobacter*

## Data Availability

The original data are included in the article. Further inquiries can be directed to the corresponding author.

## Abbreviations

rRNA, ribosomal RNA; NGS, next-generation sequencing; QCMD, Quality Control for Molecular Diagnostics; NCBI, National Center for Biotechnology Information; ASP, Advanced Sequencing Platform; USD, United States dollars.
